# Infliximab Inhibits Colitis Associated Cancer in Model Mice by Downregulating Genes Associated with Mast Cells and Decreasing Their Accumulation

**DOI:** 10.3390/cimb45040189

**Published:** 2023-04-01

**Authors:** Dan-Yang Wang, Shinobu Ohnuma, Hideyuki Suzuki, Masaharu Ishida, Kentaro Ishii, Takashi Hirosawa, Tomoaki Hirashima, Megumi Murakami, Minoru Kobayashi, Katsuyoshi Kudoh, Sho Haneda, Hiroaki Musha, Takeshi Naitoh, Michiaki Unno

**Affiliations:** Department of Surgery, Tohoku University Graduate School of Medicine, Sendai 980-8574, Japan

**Keywords:** inflammatory bowel disease, Crohn’s disease, ulcerative colitis, colitis-associated cancer, TNF-α, anti TNF-α antibody, infliximab, mast cell, azoxymethane (AOM)/dextran sodium sulfate (DSS) mouse

## Abstract

Inflammatory bowel diseases (IBDs), such as Crohn’s disease or ulcerative colitis, can be treated with anti TNF-alpha (TNF-α) antibodies (Abs), but they also put patients with IBDs at risk of cancer. We aimed to determine whether the anti TNF-α Ab induces colon cancer development in vitro and in vivo, and to identify the genes involved in colitis-associated cancer. We found that TNF-α (50 ng/mL) inhibited the proliferation, migration, and invasion of HCT8 and COLO205 colon cancer cell lines and that anti TNF-α Ab neutralized TNF-α inhibition in vitro. The effects of anti TNF-α Ab, infliximab (10 mg/kg) were investigated in mouse models of colitis-associated cancer induced by intraperitoneally injected azoxymethane (AOM: 10 mg/kg)/orally administered dextran sodium sulfate (DSS: 2.5%) (AOM/DSS) in vivo. Infliximab significantly attenuated the development of colon cancer in these mice. Microarray analyses and RT-qPCR revealed that *mast cell protease 1*, *mast cell protease 2*, and *chymase 1* were up-regulated in cancer tissue of AOM/DSS mice; however, those mast cell related genes were downregulated in cancer tissue of AOM/DSS mice with infliximab. These results suggested that mast cells play a pivotal role in the development of cancer associated with colitis in AOM/DSS mice.

## 1. Introduction

Inflammatory bowel diseases (IBDs), including Crohn’s disease (CD) and ulcerative colitis (UC), are chronic states that cause intestinal inflammation in any part of the gastrointestinal tract and in all parts of the large intestine, respectively [1,2]. The incidence and prevalence of IBDs are increasing in different regions around the world. The highest annual incidences of UC were 24.3 in Europe, 6.3 in Asia and the Middle East, and 19.2 in North America (per 100,000 person years), respectively. The highest annual incidences of CD were 12.7 in Europe, 5.0 in Asia and the Middle East, and 20.2 in North America (per 100,000 person years), respectively [3]. Uncontrolled inflammation leads to intestinal stenosis, obstruction, bleeding, and perforation in patients with IBDs, which results in high morbidity and a poor quality of life [1,2]. Therefore, effective medications to maintain the remission of IBDs are essential. IBDs are currently treated with 5-aminosalicylic acid (5-ASA), steroids, immunomodulators, antibiotics, and anti TNF-α antibodies (Abs). Among them, anti TNF-α Abs was a major breakthrough in IBD treatment [4]. Anti TNF-α Abs have reduced hospitalizations and surgical interventions in patients with IBDs [5]. Recently, other novel anti-inflammatory treatments focusing inflammatory pathways, such as cellular signaling anti-interleukin 12/23 agents and Janus kinase inhibitors and leukocyte trafficking, have been developed [2]. Furthermore, new therapeutic targets, including B cells, innate lymphoid cells, bile acids, the brain–gut axis, and microbiota have been highlighted [2]. Although treatment failure is seen in a significant proportion of patients treated with anti TNF-α Abs, anti TNF-α Abs are still mainstream to treat patients with IBDs [6].

The proinflammatory cytokine TNF-α is involved in systemic inflammation. The balance of TNF-α and other proinflammatory cytokines is maintained by anti-inflammatory factors, and disruption of this balance might lead to IBDs [7,8]. TNF-α is elevated in inflamed tissues from patients with CD [9]. Macrophages, lymphocytes, fibroblasts, keratinocytes, mast cells, dendritic cells, and tumor cells synthesize TNF-α [10], which not only induces inflammation, but also inhibits carcinogenesis by promoting apoptosis, inducing vascular endothelial cell necrosis in tumors, and activating immunocytes and tumor immunity [11,12,13]. However, TNF-α can increase carcinogenesis by activating the Wnt/β-catenin signaling pathway, inducing mutations in oncogenes, and promoting angiogenesis in tumors, as well as the proliferation and invasiveness of cancer cells [13,14,15]. Patients with CD have a 2.4-fold increased risk of colorectal cancer [16], and those with IBDs treated with anti TNF-α Abs are at risk of developing cancer [17,18,19,20]. In fact, the product label for anti TNF-α Ab warns against its application to patients with extant cancer. We encountered four cancer patients with CD and a history of anti TNF-α Ab therapy before being diagnosed with advanced anorectal cancer [18]. This raises the question of whether anti TNF-α Ab induces carcinogenesis in the colon. 

The present study analyzed the effects of TNF-α and the anti TNF-α Ab on the proliferation, migration, and invasion of human colon cancer cell lines in vitro. Then, we verified if anti TNF-α Ab inhibits carcinogenesis in mouse models of colitis-associated cancer in vivo. Furthermore, comprehensive gene expression analysis was carried out to identify the genes involved in colitis-associated cancer.

## 2. Materials and Methods

### 2.1. Chemicals and Cell Lines

The following reagents and cell lines were purchased from the respective suppliers; RPMI 1640, fetal bovine serum (FBS) and penicillin-streptomycin (Sigma-Aldrich Corp., St. Louis, MO, USA); azoxymethane (AOM; Wako Chemicals, Osaka, Japan); dextran sulfate sodium (DSS) salt (colitis-induced grade) (MP Biomedicals Inc., Irvine, CA, USA); infliximab (IFX; Mitsubishi Tanabe, Tokyo, Japan); and Human TNF-α (Abcam 9642), human anti TNF-α antibody (Abcam 7742; both from Abcam, Cambridge, UK). The colon cancer cell lines HCT8, HCT116, and COLO205 (ATCC., Manassas, VA, USA) were cultured in RPMI-1640 medium containing 10% FBS and 1% penicillin-streptomycin at 37 °C under a 5% CO_2_ atmosphere.

### 2.2. Proliferation Assays

Cell proliferation was determined by counting cells stained with Trypan blue. Cancer cells (2 × 10^4^) in 2 mL of medium were seeded in 24-well dishes and incubated for 12 h. Thereafter, 10 or 50 ng/mL of TNF-α with or without 2 µg/mL of human anti TNF-α antibody were added to fresh medium and incubated for 24 h. Then, the cells stained with 0.4% Trypan blue on slides were enumerated using a TC20 Automated Cell Counter (Bio-Rad Laboratories Inc., Hercules, CA, USA). Cell survival was calculated as ratios (%) of live cells incubated with TNF-α with or without anti TNF-α antibody to control cells. The results were obtained from three independent experiments.

### 2.3. Migration Assays

Cancer cell migration was determined using scratch assays as follows. Briefly, 2 × 10^5^ cancer cells in 2 mL media/well were seeded in 12-well dishes; then, 50 ng/mL of TNF-α and/or 2 µg/mL of human anti TNF-α antibody were added to the wells. Confluent cell monolayers were scratched using Eppendorf pipet tips. Images of scratches were captured before and at 48 h after incubation in RPMI-1640 medium without FBS. Digital images of gap closure were acquired using a BZ-9000 microscope (Keyence, Tokyo, Japan). Migration areas were analyzed using ImageJ software, and data are presented as decreases in the average migration area relative to that of controls. The results were obtained from three independent experiments.

### 2.4. Invasion Assays

Invasiveness was assayed using BioCoat Matrigel Invasion Chambers (Corning Inc., Corning, NY, USA), as described by the manufacturer. Cancer cells (1 × 10^5^) with FBS-free medium were placed in the chambers, then incubated for 22 h with 50 ng/mL of TNF-α and/or 2 µg/mL of human anti TNF-α antibody. Cells on the lower surface of the filter in the chamber were fixed and stained with 1% crystal violet (Wako, Osaka, Japan). Cell invasion was defined as density (%) in five selected microscope fields at 20 h, compared with 0 h. Data are shown as increases in average cell density relative to controls. The results were obtained from three independent experiments.

### 2.5. Animal Experiments

**Figure 1:**
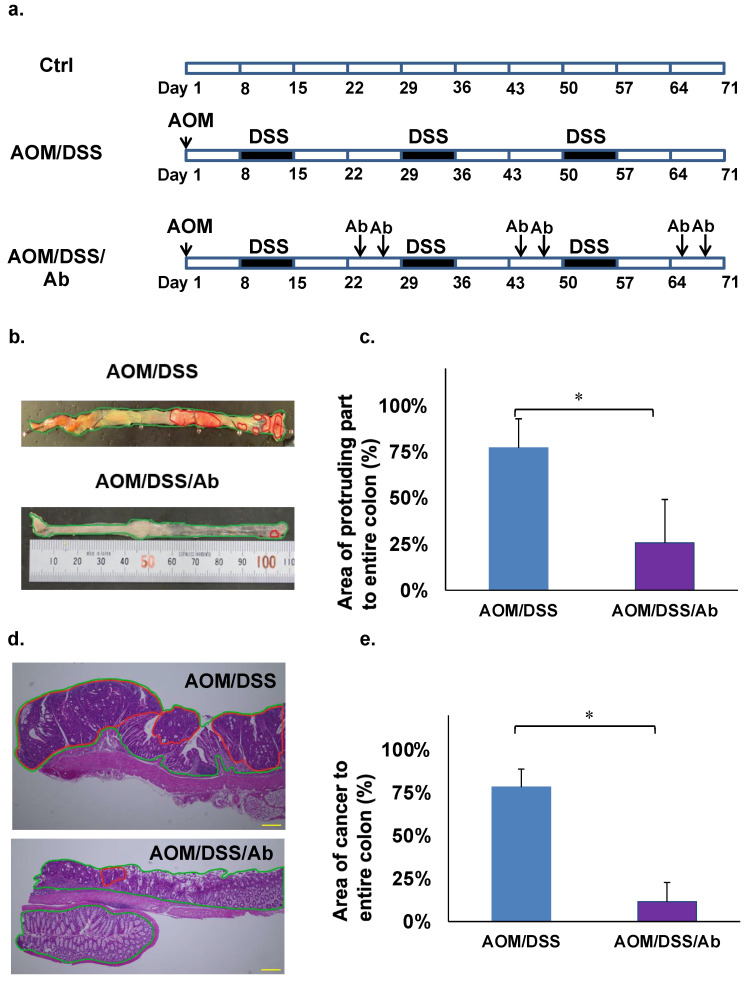
Findings of cancer associated with colitis in mouse models. (**a**) Induction of colitis-associated cancer in mice. (**b**) Macroscopic findings of colon, AOM/DSS (above), AOM/DSS/Ab (below). The areas highlighted by red circles indicate tumor-like lesions. (**c**) Comparison of protruding areas between AOM/DSS and AOM/DSS/Ab groups. (**d**) Colon cancer tissues stained with HE. AOM/DSS (above), AOM/DSS/Ab (below); The areas highlighted by red circles indicate lesions of adenocarcinoma. Scale bars represent 50 μm. (**e**) Comparison of adenocarcinoma areas between AOM/DSS and AOM/DSS/Ab groups. Ab, Infliximab; AOM, azoxymethane; DSS, dextran sodium sulfate; AOM/DSS, mice administered with AOM and DSS; AOM/DSS/Ab, mice administered with AOM, DSS, and Infliximab; Ctrl, control; HE, hematoxylin and eosin. ↓ Intraperitoneal administration. * *p* < 0.01.

The AOM/DSS model was created by injecting AOM (10 mg/kg) i.p. and DSS (2.5%) p.o. and replicates the features of human cancer associated with colitis in mice [21]. The carcinogenic rate of this model is 80–100% [21]. Five-week-old male ICR mice (CLEA Japan, Tokyo, Japan) were fed with standard commercial pellet diets and ordinary tap water and acclimated in an air-conditioned room at 24 °C for one week. The mice (n = 3 per group) were then assigned to a control group, or a group treated with AOM and DSS (AOM/DSS) or with AOM, DSS, and infliximab (AOM/DSS/Ab). Mice in both groups were injected i.p. with 10 mg/kg AOM. One week later, the mice started on three cycles of orally administered 2.5% DSS in tap water for one week, followed by 2 weeks of recovery (Figure 1a). The AOM/DSS/Ab group was also injected i.p. twice with 10 mg/kg infliximab in 200 μL of saline during the third week of each cycle (Figure 1a). The mice were weighed, and fecal consistency was evaluated daily. All mice were euthanized at the end of the third cycle, then the entire colon was removed and cut into seven pieces. Portions with cancer and corresponding normal tissues were stored in liquid nitrogen, and the remainder was fixed in 10% formalin for 2 days and processed into paraffin blocks for histological analyses. All animal studies proceeded under strict ethical considerations for the use of experimental animals according to the Guidelines for the Care and Use of Laboratory Animals of Tohoku University. The Animal Care and Use Committee of the Tohoku University Graduate School of Medicine approved all animal care procedures (Approval ID: 2017-173).

### 2.6. Calculation of Cancerous Areas in the Colon

Photographs of whole colons from euthanized mice were stored on a computer. The area of the protruding part of the colon that might include cancer in the images was macroscopically examined by an animal pathologist. All tumor-like parts of the colon were macroscopically counted, and areas of tumor-like lesions were calculated using Image J (http://rsb.info.nih.gov/ij/, accessed on 29 August 2017) (Figure 1b,c). After confirming colon cancer in HE-stained slides, areas of cancerous lesions were histologically validated and calculated as (area of cancer/area of entire colon) × 100 (%) using Image J (Figure 1d,e).

### 2.7. Microarray Analysis

Total RNA extracted from control, normal and cancerous tissues of AOM/DSS and AOM/DSS/Ab mice were analyzed using a 3D-Gene Mouse Oligo chip 24k microarray (Toray Industries Inc., Tokyo, Japan). Total RNA was labeled with Cy5 using Amino Allyl MessageAMP^TM^ II aRNA Amplification Kits (Thermo Fisher Scientific Inc., Waltham, MA, USA) and hybridized for 16 h, as described by the manufacturer (www.3d-gene.com, accessed on 7 September 2017). Hybridization signals obtained using a 3D-Gene Scanner were processed using 3D-Gene Extraction (Both from Toray Industries Inc.). Signals for genes of interest were globally normalized to an adjusted median intensity of 25.

### 2.8. Staining Mast Cells with Toluidine Blue 

Mast cells in colon tissues were detected by HCL-Toluidine blue staining. Tissue sections on slides were dewaxed, washed with distilled water, then stained at room temperature for 30 min in 0.5 N HCL containing 0.5% powdered Toluidine blue. Tissue sections were washed with distilled water, dried, and coverslipped using xylene, then an animal pathologist examined the specimens using a microscope. Mast cells were calculated as the number per area of the entire colon as follows: the positively stained mast cells in the sample tissues were detected by selecting seven random fields in high-power microscope (×400) after microscopic examination scanning the entire colon tissues in three mice. The mast cell counts are the mean of seven individual high-power fields.

### 2.9. Reverse Transcription Quantitative PCR (RT-qPCR)

Total RNA (0.5 μg) was transcribed into cDNA using PrimeScript RT Reagent Kits (Takara Bio) as described by the manufacturer, then RT-qPCR proceeded using the StepOnePlus Real-Time PCR system with FAST SYBR Green Master Mix (both from Thermo Fisher Scientific Inc.). Messenger RNA expression in samples was relatively quantified using the 2^−ΔΔCt^ method, and the results were normalized against those of the internal control mouse β-actin. The forward and reverse (5′ → 3′) primers were as follows: 

Mus-Mcpt1: TTCCAGGTCTGTGTGGGAAG and TCCAGGGCACATATGCAGAG,

Mus-Mcpt2: AACGGTTCGAAGGAGAGGTG and TCTGCTGTGTGGGTTCGTTC,

Mus-Cma1 (chymase 1, mast cells): ATAAGCCTAAGGCCCAAATATGA and CAATGATCTCTCCAGCTTTGGT, 

β-actin: GGCTGTATTCCCCTCCATCG and CCAGTTGGTAACAATGCCATGT [22].

### 2.10. Statistical Analysis

Data are shown as the means ± standard error (SE) of at least three independent experiments. Differences between means were analyzed using Student *t*-tests. Values with *p* < 0.05 were considered statistically significant. All data were statistically analyzed using JMP v.12.2 (SAS International Inc., Cary, NC, USA).

## 3. Results

### 3.1. Proliferation Assays

**Figure 2:**
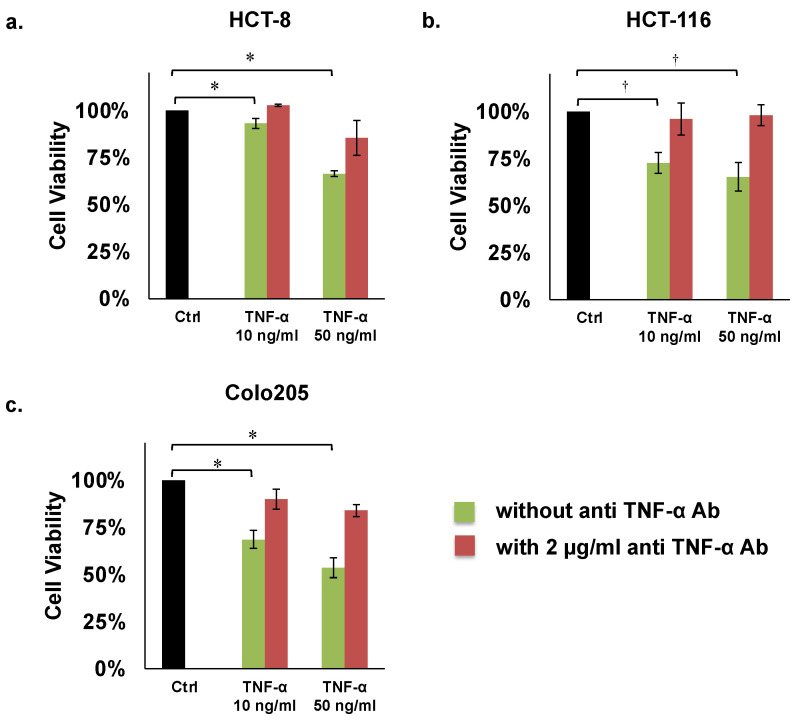
Proliferation assays of colon cancer cells. (**a**) HCT8, (**b**) HCT116, (**c**) COLO205. Concentrations of TNF-α: 10 or 50 ng/mL. Concentration of human anti TNF-α Ab: 2 µg/mL. * *p* < 0.01, ^†^ *p* < 0.05.

The results of these assays revealed that human TNF-α (50 ng/mL) significantly inhibited proliferation of the HCT8, HCT116, and COLO205 colon cancer cell lines by 66%, 65%, and 54%, respectively, compared with controls (Figure 2a–c; Table 1). However, TNF-α mediated inhibition of these three cell lines was recovered by adding 2 µg/mL of human anti TNF-α Ab (Figure 2a–c; Table 1).

### 3.2. Migration Assays

**Figure 3:**
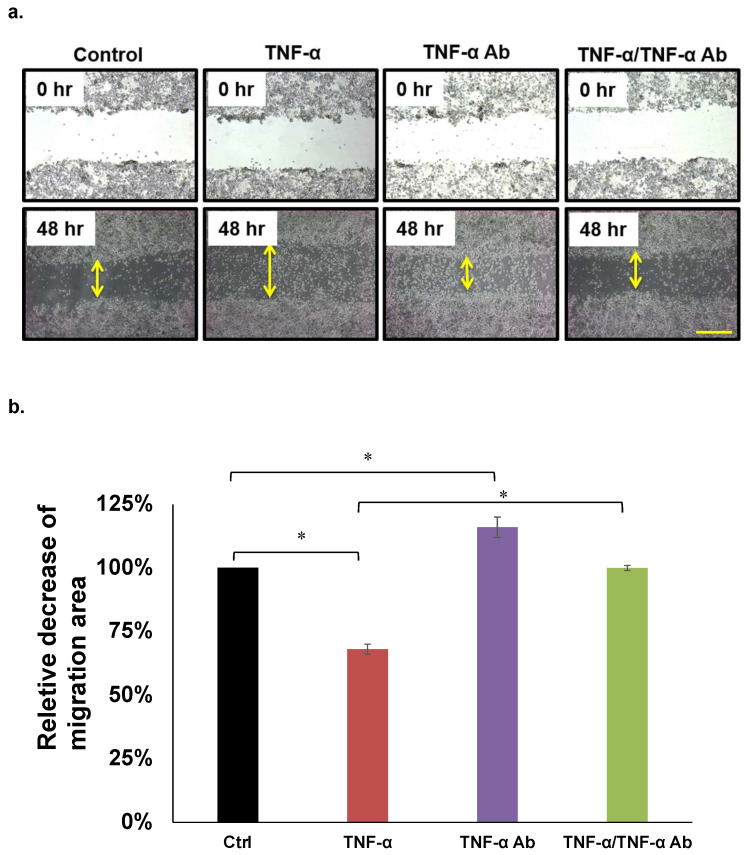
Migration assays. (**a**,**b**) Representative COLO205 data. Concentrations of TNF-α and human anti TNF-α Ab: 50 ng/mL and 2 µg/mL, respectively. * *p* < 0.05. Scale bar represents 200 μm.

We analyzed the effects of TNF-α on HCT8, HCT116, and COLO205 cell migration using wound healing assays. Figure 2a shows that 50 ng/mL of TNF-α significantly attenuated, whereas human anti TNF-α Ab (2 µg/mL) recovered the migration of COLO205 cells (Figure 3a,b). This trend was similar in HCT8, but not in HCT116 cells (Table 1).

### 3.3. Invasion Assays

**Figure 4:**
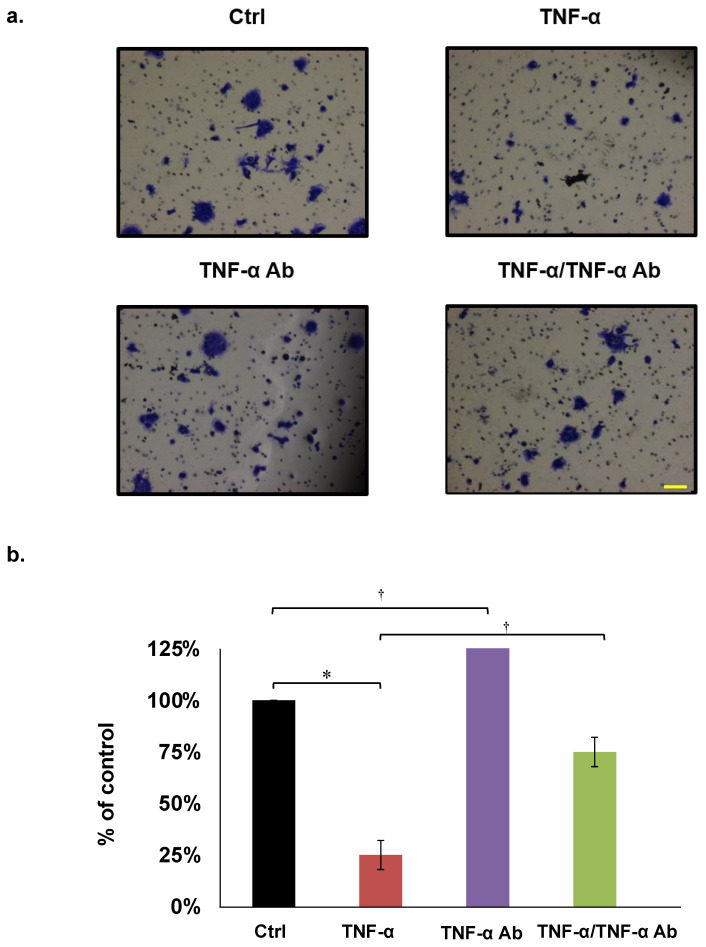
Invasion assays. (**a**,**b**) Representative COLO205 data. Concentrations of TNF-α and human anti TNF-α Ab are 50 ng/mL and 2 µg/mL, respectively. * *p* < 0.01, ^†^ *p* < 0.05. Scale bar represents 100 μm.

We assayed the effects of TNF-α on HCT8, HCT116, and COLO205 cell invasion using Matrigel chambers. The results showed that TNF-α (50 ng/mL) significantly inhibited, whereas human anti TNF-α Ab (2 µg/mL) neutralized COLO205 invasion (Figure 4a,b). These trends were similar in HCT8 and HCT116 cells (Table 1).

### 3.4. Effects of Infliximab in Azoxymethane/Dextran Sodium Sulfate (AOM/DSS) Model Mice

The body weight decreased by 10–20% in azoxymethane (AOM)/dextran sodium sulfate (DSS) mice and in those treated with anti TNF-α Ab infliximab (AOM/DSS/Ab), and both groups also developed diarrhea and rectal bleeding when administered with DSS water (data not shown). Neoplastic lesions were found in the colons of all euthanized mice, but significantly fewer were evident in AOM/DSS/Ab, than AOM/DSS mice (Figure 1b). Tumor-like lesions occupied 71.7% and 25.7% of colons from AOM/DSS and AOM/DSS/Ab mice, respectively, and differed significantly (*p* < 0.01; Figure 1c). Staining with hematoxylin and eosin (HE) revealed that adenocarcinoma accounted for 78.2% and 11.5% of the areas with colon cancer lesions in the AOM/DSS and AOM/DSS/Ab mice, respectively (*p* < 0.01; Figure 1d,e). 

### 3.5. Microarray Analysis

Because infliximab significantly decreased the incidence of colon cancer in AOM/DSS mice, we compared downregulated genes in cancer tissues between the AOM/DSS/Ab and AOM/DSS mice. We identified 100 genes that were upregulated in cancer tissues of the AOM/DSS group compared with normal control tissues. We then compared downregulated genes in cancer tissues from the AOM/DSS/Ab and AOM/DSS groups. Table 2 shows the top 10 downregulated genes including *mast cell proteases 1*,* 2*,* and 4* (*Mctp1*,* Mctp2*,* Mctp4*), *chymase 1* (*Cma1*), *Fc receptor IgE high affinity I alpha polypeptide* (*Fcer1a*), *phospholipase A2*, *group IIE* (*Pla2g2e*), and *carboxypeptidase A3* (*Cpa3*). All these genes were associated with mast cells. 

### 3.6. Toluidine Blue Staining

**Figure 5:**
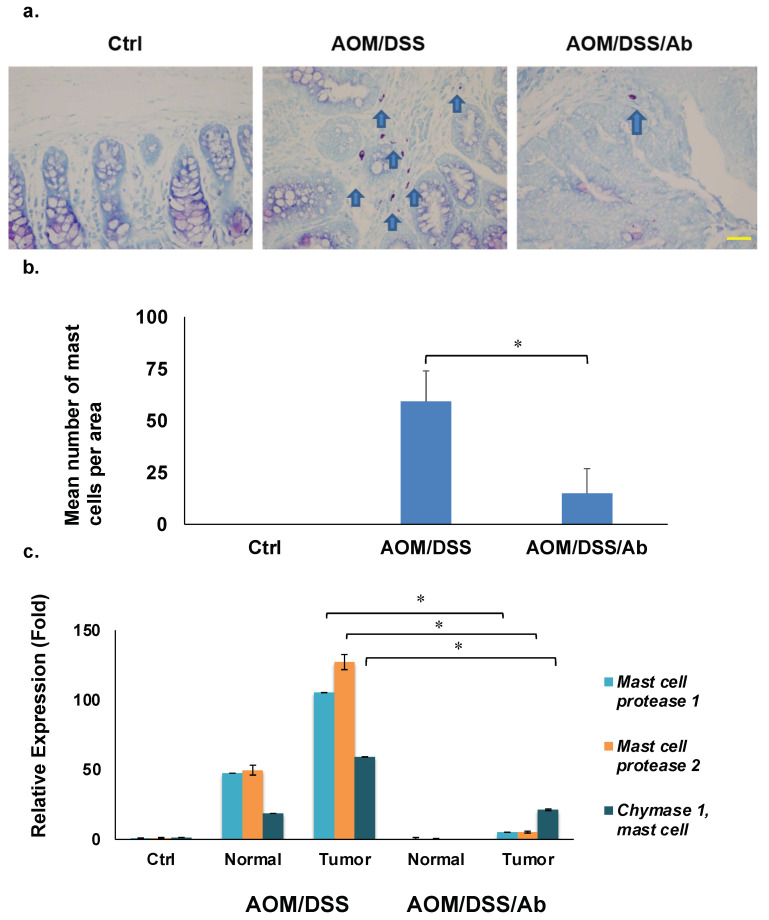
Results of staining and quantifying mast cells and RT-qPCR. (**a**) Mast cells stained with HCL-Toluidine blue (magnification: ×400). Scale bar represents 50 μm. (**b**) Comparison of mast cell numbers. (**c**) Results of RT-qPCR of *mast cell protease 1*, *mast cell protease 2*, and *chymase 1*, * *p* < 0.01. Ab, Infliximab; AOM, azoxymethane; AOM/DSS, mice administered with AOM and DSS; AOM/DSS/Ab, mice administered with AOM, DSS, and infliximab; Ctrl, control; DSS, dextran sodium sulfate; normal, normal tissue; tumor, cancer tissue.

Staining with HCL-Toluidine revealed that infliximab significantly decreased the high abundance of mast cells in colon cancer tissues from AOM/DSS mice (59.2 vs. 14.9, *p* < 0.01). (Figure 5a,b).

### 3.7. Reverse Transcription Quantitative PCR (RT-qPCR)

Table 2 shows several downregulated mast cell-related genes in the AOM/DSS/Ab group. We analyzed expression of the top three genes, *Mcpt1*, *Mcpt2*, and *Cma1* using RT-qPCR to validate the microarray results. The mRNA expression of these genes was 100-, 125-, and 60-fold higher, respectively, in cancer tissues from AOM/DSS, than control mice, and infliximab significantly inhibited their expression compared to the control 5-, 5-, and 21-fold, respectively (*p* < 0.01; Figure 5c).

## 4. Discussion

This study found that TNF-α inhibited the proliferation, migration, and invasion of human colon cancer cells, and that this was offset by anti TNF-α Ab in vitro. In contrast, infliximab significantly inhibited colon cancer development in AOM/DSS mice in vivo.

Although TNF-α is rarely cytotoxic against cancer cells in vitro [23] the present findings revealed that TNF-α inhibited colon cancer cell growth and motility. Although TNF-α induces apoptosis in colon cancer cells [24], it can also induce the proliferation and migration of colon cancer cell lines [25]. The growth of BxPc3 and COLO 357 pancreatic cancer cells similarly reduced by TNF-α, but it also strongly enhances their invasive properties [26]. Therefore, diverse functions of TNF-α in cell signaling are hardly surprising.

Cell signaling starts after TNF-α binds to TNF-α receptor 1 (TNFR1), which then sequentially recruits TNFR-associated death domain (TRADD), TNFR-associated factor 2 (TRAF2), receptor-interacting protein (RIP), and inhibitor of nuclear factor-kappa B (NF-κB) kinase (IKK). This cascade leads to NF-κB activation which induces inflammatory cytokines and anti-apoptotic proteins. In contrast, the recruitment of TRADD, FAS-associated death domain (FADD) and caspase-8 leads to caspase-3 activation, which in subsequently induces apoptosis [23]. Therefore, cell proliferation, migration, and invasive properties might be altered in vitro depending on dominant cell signaling mediated by TNF-α in various cell lines.

Orally administered DSS induces inflammation and ulceration in the mouse colon, and the intraperitoneal (i.p). administration of AOM leads to the development of aberrant crypt foci (ACF) in the colonic mucosa, which causes the emergence of colon cancer [21]. Therefore, the AOM/DSS mouse models are useful tools for analyzing colitis-associated carcinogenesis and detecting modulators [27].

Our findings in vivo showed that infliximab significantly inhibited colon cancer development in AOM/DSS mice. Figure 4c shows that adenocarcinoma occupied 78.2% and 11.5% of colonic areas in the AOM/DSS and AOM/DSS/Ab mice, respectively. Infliximab attenuated colon cancer development by 85%. Inflammatory cells such as granulocytes and macrophages in the inflamed mucosa of AOM/DSS mice upregulate TNF-α expression [28], which then activates NF-κB in intestinal epithelial cells and promotes tumor growth [29,30]. Moreover, etanercept (a TNF-α blocker) inhibits colon carcinogenesis in AOM/DSS mice by decreasing levels of prostaglandin H2 and E2 secreted by inflammatory cells, which are essential mediators of angiogenesis [31], and by inhibiting the Wnt/B-catenin signaling pathway that is associated with colon carcinogenesis [28]. Therefore, the direct inhibition of TNF-α by infliximab reduced colon cancer formation in the AOM/DSS/Ab mice.

Our microarray data provided new evidence that mast cells play roles in colitis-associated cancer in AOM/DSS mice. Microarray analysis of cancer tissues from AOM/DSS and AOM/DSS/Ab mice revealed profound differences in the expression of genes associated with mast cells, namely *Mctp1*, *Mctp2*, *Mctp4*, *Cma1*, *Fcer1a*, *Pla2g2e*, and *Cpa3*. The top three genes, *Mctp1*, *Mctp2*, and *Cma1* were validated by RT-qPCR, which confirmed the microarray results. Furthermore, Toluidine blue staining confirmed an increased abundance of mast cells in interstitial tissues of the AOM/DSS group. Collectively, these results suggested that the development of colitis-associated cancer is associated with mast cell accumulation in the mouse colon. 

Mast cells are involved in IBDs. The numbers of mast cells and of those positive for chymase are increased in the intestinal mucosa of patients with active CD [32]. Chemical mediators such as histamine and tryptase are released from mast cells in patients with IBD [33,34]. Mast cells are bone-marrow-derived multifunctional immune cells that have secretory granules that contain proteases, chymase, tryptase, and carboxypeptidase A3 and were recognized by Paul Ehrlich 130 years ago [35]. Mast cell proteases significantly influence not only inflammation but also angiogenesis, thus affecting tumor growth and progression by acting on immunosuppression, release of the proangiogenic cytokines VEGF, IL-18, and TNF-α, mitogenic factors, and extracellular matrix degradation [36,37]. Mast cells are also an important source of TNF-α [38,39], which further induces mast cell accumulation in tissues, which can be blocked by infliximab [40]. Inflammation-induced cytokines play pivotal roles in the initiation, promotion, and progression of colon carcinogenesis [41]. Our in vivo results suggested that anti TNF-α Ab, infliximab could inhibit mast cell accumulation in the colon, resulting in decreased levels of TNF-α and attenuated colon cancer in AOM/DSS mice. Moreover, inflammatory cells such as mast cells in the colon might explain the different assay results of cancer progression between in vitro and in vivo. Therefore, anti TNF-α Ab did not inhibit colon cancer cell lines in vitro, but inhibited colon cancer development in mice in vivo. Although mast cells may play pivotal roles in the development of colitis-associated cancer, only the data of mRNA expressions were shown in this study. To prove the roles of mast cells in colitis-associated cancers, further experiments are needed, for example, an experiment to see the role of mast cells during colitis with or without infliximab treatment using a mast cell-deficient mouse model [42].

One limitation of this study is that we used infliximab, which was designed for humans, in experiments involving mice. Infliximab exerts considerable anti-inflammatory or anti-cancer effects, even in mouse models [43]. However, to evaluate the precise mechanism of infliximab in colon carcinogenesis in mice, an anti-mouse TNF-α Ab might require consideration [29]. Furthermore, we injected the mice with infliximab i.p., to ensure adequate coverage, whereas patients with CD are injected intravenously. Therefore, intravenous infliximab should also be considered in future studies of experimental animals.

## 5. Conclusions

This study revealed that anti TNF-α Ab infliximab significantly inhibited colon carcinogenesis in AOM/DSS mice. Furthermore, mast cell accumulation and the mRNA expression of genes associated with mast cells were significantly decreased in colon cancer tissues from mice treated with infliximab. These findings indicated that mast cells play pivotal roles in the development of colitis-associated cancer. The precise roles of mast cells and TNF-α in IBDs and colitis-associated cancer have not been fully elucidated. Further investigation is warranted to determine whether infliximab could prevent colonic mast cell accumulation and colitis-associated cancer in patients with IBDs.

## Figures and Tables

**Table 1:** Summary of proliferation, migration, and invasion assays of colon cancer cell lines incubated with TNF-α and anti TNF-α Ab.

Assays	Cell Lines	Inhibition by TNF-α (50 ng/mL)	Recovery Effect by Anti TNF-α Ab (2 µg/mL)
Proliferation	HCT8	66% *	85%
	HCT116	65% ^†^	98%
	COLO205	54% *	84%
Migration	HCT8	74% ^†^	91%
	HCT116	None	None
	COLO205	61% ^†^	100%
Invasion	HCT8	49% *	99%
	HCT116	12% *	20%
	COLO205	23% *	75%

Values are shown as ratios to control set at 100%. * *p* < 0.01, ^†^ *p* < 0.05.

**Table 2:** Microarray results of top 10 downregulated genes in cancerous tissues from AOM/DSS mice with and without anti TNF-α Ab.

Gene	Name	Ab(-)/Normal *	Ab(-)/Ab(+) ^†^
*Mcpt1*	*Mast cell protease 1*	58.62	8.77
*Mcpt2*	*Mast cell protease 2*	54.93	7.91
*Cma1*	*Chymase 1*,* mast cell*	71.01	4.84
*Mcpt4*	*Mast cell protease 4*	17.21	4.76
*Fcer1a*	*Fc receptor*,* IgE*,* high affinity I*,* alpha polypeptide*	27.10	4.74
*9530003J23Rik*	*RIKEN cDNA 9530003J23*	17.13	4.32
*Pla2g2e*	*Phospholipase A2*,* group IIE*	19.44	4.21
*Mmp8*	*Matrix metallopeptidase 8*	15.23	3.20
*Cpa3*	*Carboxypeptidase A3*,* mast cell*	13.30	2.79
*Krt35*	*Keratin 35*	122.87	2.78

The top 10 genes downregulated in cancer tissue of AOM/DSS mice by adding TNF-α Ab are shown with gene symbols. * Ab (-)/Normal, Values were calculated by dividing relative gene expression in cancer of AOM/DSS (Ab-) by that in normal control (Normal). ^†^ Ab (-)/Ab (+), Values were calculated by dividing relative gene expression in cancer of AOM/DSS (Ab(-)) by that of AOM/DSS/Ab (Ab(+)).
